# Supplementary material for: Plasma proteomics reveals markers of metabolic stress in HIV infected children with severe acute malnutrition
Source: Sci Rep. 2020 Jul 8;10:11235. doi: 10.1038/s41598-020-68143-7 (PMC7343797; doi:10.1038/s41598-020-68143-7)
Supplement: Supplementary file 1 — Supplementary Information 1 (DOCX 112 kb) [file 41598_2020_68143_MOESM1_ESM.docx]

SUPPLEMENTARY FILE

Plasma proteomics reveals markers of metabolic stress in HIV infected children with severe acute malnutrition

Gerard Bryan Gonzales^1,2*^, James M Njunge^3,4^, Bonface M Gichuki^3,4^, Bijun Wen^5,6^, Isabel Potani^3^, Wieger Voskuijl^3,7,8^, Robert H J Bandsma^3,5,6^, James A Berkley^3,4,9^

1. Department of Gastroenterology, Faculty of Medicine and Health Sciences, Ghent University, Ghent, Belgium
2. VIB Inflammation Research Centre, Ghent, Belgium
3. The Childhood Acute Illness & Nutrition (CHAIN) Network, Nairobi, Kenya
4. KEMRI/Wellcome Trust Research Programme, Kilifi, Kenya
5. Centre for Global Child Health, The Hospital for Sick Children, Toronto, Ontario, Canada
6. Department of Nutritional Sciences, Faculty of Medicine, University of Toronto, Toronto, Canada
7. Global Child Health Group, Emma Children's Hospital, Amsterdam University Medical Centres, Amsterdam, the Netherlands
8. Amsterdam Institute for Global Health and Development, Department of Global Health, Amsterdam University Medical Centres, Amsterdam, The Netherlands
9. Centre for Tropical Medicine & Global Health, Nuffield Department of Medicine, University of Oxford, Oxford, UK

Supplementary Table 1. Prior use of anti-retroviral treatment among all HIV (+) children included in the clinical trial

|  | **Prior HIV treatment*** | | | | |
| --- | --- | --- | --- | --- | --- |
|  | **HAART** | **NVP only** | **ART naïve** | **Unknown** |  |
| n (%)° | 53 (30%) | 7 (4%) | 90 (50%) | 29 (16%) |  |
| Mean MUAC at admission (cm) [95% CI] | 10.9 [10.5 - 11.4] | 9.8 [8.9 - 10.6] | 10.5 [10.2 - 10.8] | 9.6 [8.1 - 11.1] |  |
| Mortality n (%) | 10 (18%) | 1 (28%) | 25 (28%) | 1 (25%) |  |
| Odds ratio for mortality [95% CI] | 0.97 [0.82 - 1.12]  p=0.63*^#^* | - | *Reference* | 1.07 [0.88 – 1.30]  p=0.45*^#^* |  |

*HAART - highly active antiretroviral therapy; NVP – Nevirapine; *^#^* adjusted for age, sex, site and oedema

Supplementary Table 2. Association between individual proteins to HIV status

| Uniprot accession | Protein code | Protein names | log FC* | *p*° |
| --- | --- | --- | --- | --- |
| P05109 | S100A8 | Protein S100-A8 (Calgranulin-A) (Calprotectin L1L subunit) (Cystic fibrosis antigen) (CFAG) (Leukocyte L1 complex light chain) (Migration inhibitory factor-related protein 8) (MRP-8) (p8) (S100 calcium-binding protein A8) (Urinary stone protein band A) | 0.56 | 0.00 |
| P06702 | S100A9 | Protein S100-A9 (Calgranulin-B) (Calprotectin L1H subunit) (Leukocyte L1 complex heavy chain) (Migration inhibitory factor-related protein 14) (MRP-14) (p14) (S100 calcium-binding protein A9) | 0.55 | 0.00 |
| P01708 |  | Immunoglobulin lambda variable 2-11 (Ig gamma lambda chain V-II region DOT) (Ig lambda chain V-II region BOH) (Ig lambda chain V-II region BUR) (Ig lambda chain V-II region NIG-58) (Ig lambda chain V-II region TRO) (Ig lambda chain V-II region WIN) | 0.59 | 0.01 |
| P04275 | VWF | von Willebrand factor (vWF) [Cleaved into: von Willebrand antigen 2 (von Willebrand antigen II)] | 0.33 | 0.01 |
| P01880 | IGHD | Immunoglobulin heavy constant delta (Ig delta chain C region) (Ig delta chain C region NIG-65) (Ig delta chain C region WAH) | 0.60 | 0.01 |
| P23083 | IGHV1-2 | Immunoglobulin heavy variable 1-2 (Ig heavy chain V-I region ND) (Ig heavy chain V-I region V35) | 0.60 | 0.01 |
| P01598 |  | Immunoglobulin kappa variable 1-5 (Ig kappa chain V-I region CAR) (Ig kappa chain V-I region EU) (Ig kappa chain V-I region HK102) (Ig kappa chain V-I region Kue) | 0.55 | 0.01 |
| P80748 | IGLV3-21 | Immunoglobulin lambda variable 3-21 (Ig lambda chain V-III region LOI) (Ig lambda chain V-V region DEL) (Ig lambda chain V-VII region MOT) | 0.58 | 0.01 |
| P13796 | LCP1 | Plastin-2 (L-plastin) (LC64P) (Lymphocyte cytosolic protein 1) (LCP-1) | 0.28 | 0.01 |
| P01857 | IGHG1 | Immunoglobulin heavy constant gamma 1 (Ig gamma-1 chain C region) (Ig gamma-1 chain C region EU) (Ig gamma-1 chain C region KOL) (Ig gamma-1 chain C region NIE) | 0.64 | 0.01 |
| P05362 | ICAM1 | Intercellular adhesion molecule 1 (ICAM-1) (Major group rhinovirus receptor) (CD antigen CD54) | 0.28 | 0.01 |
| P06318 |  | Immunoglobulin lambda variable 6-57 (Ig lambda chain V-VI region AR) (Ig lambda chain V-VI region EB4) (Ig lambda chain V-VI region NIG-48) (Ig lambda chain V-VI region SUT) (Ig lambda chain V-VI region WLT) | 0.49 | 0.01 |
| P18428 | LBP | Lipopolysaccharide-binding protein (LBP) | 0.31 | 0.01 |
| P01834 | IGKC | Immunoglobulin kappa constant (Ig kappa chain C region) (Ig kappa chain C region AG) (Ig kappa chain C region CUM) (Ig kappa chain C region EU) (Ig kappa chain C region OU) (Ig kappa chain C region ROY) (Ig kappa chain C region TI) | 0.69 | 0.02 |
| P0CG05 |  | Immunoglobulin lambda constant 3 (Ig lambda chain C region DOT) (Ig lambda chain C region NEWM) (Ig lambda-3 chain C regions) | 0.67 | 0.02 |
| P0CG05 |  | Immunoglobulin lambda constant 2 (Ig lambda chain C region Kern) (Ig lambda chain C region NIG-64) (Ig lambda chain C region SH) (Ig lambda chain C region X) (Ig lambda-2 chain C region) | 0.67 | 0.02 |
| B9A064 | IGLL5 | Immunoglobulin lambda-like polypeptide 5 (G lambda-1) (Germline immunoglobulin lambda 1) | 0.60 | 0.02 |
| H9KV70 |  | Deleted. | 0.27 | 0.02 |
| P01597 | IGKV1-39 | Immunoglobulin kappa variable 1-39 (Ig kappa chain V-I region DEE) (Ig kappa chain V-I region Hau) (Ig kappa chain V-I region Mev) (Ig kappa chain V-I region OU) (Ig kappa chain V-I region Walker) | 0.47 | 0.02 |
| P01714 | IGLV3-19 | Immunoglobulin lambda variable 3-19 (Ig lambda chain V-III region SH) | 0.54 | 0.02 |
| P06331 | IGHV4-34 | Immunoglobulin heavy variable 4-34 (Ig heavy chain V-II region ARH-77) | 0.49 | 0.02 |
| P19652 | ORM2 | Alpha-1-acid glycoprotein 2 (AGP 2) (Orosomucoid-2) (OMD 2) | 0.20 | 0.02 |
| P01610 |  | Immunoglobulin kappa variable 1-17 (Ig kappa chain V-I region Gal) (Ig kappa chain V-I region WEA) | 0.52 | 0.02 |
| P01781 |  | Immunoglobulin heavy variable 3-7 (Ig heavy chain V-III region GAL) (Ig heavy chain V-III region GAR) (Ig heavy chain V-III region JON) | 0.55 | 0.02 |
| P02741 | CRP | C-reactive protein [Cleaved into: C-reactive protein(1-205)] | 0.51 | 0.02 |
| P01717 | IGLV3-25 | Immunoglobulin lambda variable 3-25 (Ig lambda chain V-IV region Hil) | 0.46 | 0.02 |
| P01719 |  | Immunoglobulin lambda variable 3-21 (Ig lambda chain V-III region LOI) (Ig lambda chain V-V region DEL) (Ig lambda chain V-VII region MOT) | 0.44 | 0.02 |
| P06681 | C2 | Complement C2 (EC 3.4.21.43) (C3/C5 convertase) [Cleaved into: Complement C2b fragment; Complement C2a fragment] | 0.15 | 0.02 |
| Q15848 | ADIPOQ | Adiponectin (30 kDa adipocyte complement-related protein) (Adipocyte complement-related 30 kDa protein) (ACRP30) (Adipocyte, C1q and collagen domain-containing protein) (Adipose most abundant gene transcript 1 protein) (apM-1) (Gelatin-binding protein) | -0.23 | 0.02 |
| P04208 |  | Immunoglobulin lambda variable 1-47 (Ig lambda chain V-I region HA) (Ig lambda chain V-I region WAH) | 0.53 | 0.02 |
| Q15485 | FCN2 | Ficolin-2 (37 kDa elastin-binding protein) (Collagen/fibrinogen domain-containing protein 2) (EBP-37) (Ficolin-B) (Ficolin-beta) (Hucolin) (L-ficolin) (Serum lectin p35) | 0.26 | 0.03 |
| P18135 |  | Immunoglobulin kappa variable 3-20 (Ig kappa chain V-III region B6) (Ig kappa chain V-III region GOL) (Ig kappa chain V-III region HAH) (Ig kappa chain V-III region HIC) (Ig kappa chain V-III region IARC/BL41) (Ig kappa chain V-III region NG9) (Ig kappa chain V-III region SIE) (Ig kappa chain V-III region Ti) (Ig kappa chain V-III region WOL) | 0.57 | 0.03 |
| P01613 |  | Immunoglobulin kappa variable 1D-33 (Ig kappa chain V-I region AG) (Ig kappa chain V-I region Bi) (Ig kappa chain V-I region Lay) (Ig kappa chain V-I region Ni) (Ig kappa chain V-I region Rei) (Ig kappa chain V-I region Roy) (Ig kappa chain V-I region Scw) (Ig kappa chain V-I region WAT) | 0.45 | 0.04 |
| P01602 | IGKV1-5 | Immunoglobulin kappa variable 1-5 (Ig kappa chain V-I region CAR) (Ig kappa chain V-I region EU) (Ig kappa chain V-I region HK102) (Ig kappa chain V-I region Kue) | 0.55 | 0.04 |
| P04211 | IGLV7-43 | Immunoglobulin lambda variable 7-43 (Ig lambda chain V region 4A) | 0.49 | 0.04 |
| P01042_2 | KNG1 | Kininogen-1 (Alpha-2-thiol proteinase inhibitor) (Fitzgerald factor) (High molecular weight kininogen) (HMWK) (Williams-Fitzgerald-Flaujeac factor) [Cleaved into: Kininogen-1 heavy chain; T-kinin (Ile-Ser-Bradykinin); Bradykinin (Kallidin I); Lysyl-bradykinin (Kallidin II); Kininogen-1 light chain; Low molecular weight growth-promoting factor] | -0.28 | 0.04 |
| H0YGL9 |  | Deleted. | 0.82 | 0.04 |
| A0M8Q6 | IGLC7 | Immunoglobulin lambda constant 7 (Ig lambda-7 chain C region) | 0.54 | 0.05 |
| P01617 |  | Immunoglobulin kappa variable 2D-28 (Ig kappa chain V-II region FR) (Ig kappa chain V-II region GM607) (Ig kappa chain V-II region MIL) (Ig kappa chain V-II region TEW) | 0.37 | 0.05 |
| P01620 |  | Immunoglobulin kappa variable 3-20 (Ig kappa chain V-III region B6) (Ig kappa chain V-III region GOL) (Ig kappa chain V-III region HAH) (Ig kappa chain V-III region HIC) (Ig kappa chain V-III region IARC/BL41) (Ig kappa chain V-III region NG9) (Ig kappa chain V-III region SIE) (Ig kappa chain V-III region Ti) (Ig kappa chain V-III region WOL) | 0.44 | 0.05 |
| P01702 |  | Immunoglobulin lambda variable 1-51 (Ig lambda chain V-I region BL2) (Ig lambda chain V-I region EPS) (Ig lambda chain V-I region NEW) (Ig lambda chain V-I region NIG-64) | 0.65 | 0.05 |
| Q6UXB8_2 | PI-16 | Peptidase inhibitor 16 (PI-16) (Cysteine-rich secretory protein 9) (CRISP-9) (PSP94-binding protein) (CD antigen CD364) | -0.22 | 0.05 |
| P01876 | IGHA1 | Immunoglobulin heavy constant alpha 1 (Ig alpha-1 chain C region) (Ig alpha-1 chain C region BUR) (Ig alpha-1 chain C region TRO) | 0.51 | 0.05 |
| P04196 | HRG | Histidine-rich glycoprotein (Histidine-proline-rich glycoprotein) (HPRG) | -0.26 | 0.05 |
| P04433 | IGKV3-11 | Immunoglobulin kappa variable 3-11 (Ig kappa chain V-III region VG) | 0.57 | 0.05 |
| P06310 | IGKV2-30 | Immunoglobulin kappa variable 2-30 (Ig kappa chain V-II region RPMI 6410) | 0.52 | 0.05 |
| G8JLA8 |  | Deleted. | 0.11 | 0.07 |
| P01034 | CST3 | Cystatin-C (Cystatin-3) (Gamma-trace) (Neuroendocrine basic polypeptide) (Post-gamma-globulin) | 0.16 | 0.07 |
| P01765 |  | Immunoglobulin heavy variable 3-23 (Ig heavy chain V-III region LAY) (Ig heavy chain V-III region POM) (Ig heavy chain V-III region TEI) (Ig heavy chain V-III region TIL) (Ig heavy chain V-III region TUR) (Ig heavy chain V-III region VH26) (Ig heavy chain V-III region WAS) (Ig heavy chain V-III region ZAP) | 0.49 | 0.07 |
| P04003 | C4BPA | C4b-binding protein alpha chain (C4bp) (Proline-rich protein) (PRP) | 0.10 | 0.07 |
| P01743 | IGHV1-46 | Immunoglobulin heavy variable 1-46 (Ig heavy chain V-I region DOT) (Ig heavy chain V-I region HG3) (Ig heavy chain V-I region Mot) | 0.45 | 0.07 |
| P01031 | C5 | Complement C5 (C3 and PZP-like alpha-2-macroglobulin domain-containing protein 4) [Cleaved into: Complement C5 beta chain; Complement C5 alpha chain; C5a anaphylatoxin; Complement C5 alpha' chain] | 0.07 | 0.07 |
| P01764 | IGHV3-23 | Immunoglobulin heavy variable 3-23 (Ig heavy chain V-III region LAY) (Ig heavy chain V-III region POM) (Ig heavy chain V-III region TEI) (Ig heavy chain V-III region TIL) (Ig heavy chain V-III region TUR) (Ig heavy chain V-III region VH26) (Ig heavy chain V-III region WAS) (Ig heavy chain V-III region ZAP) | 0.49 | 0.07 |
| P01766 | IGHV3-13 | Immunoglobulin heavy variable 3-13 (Ig heavy chain V-III region BRO) | 0.41 | 0.07 |
| P01767 | IGHV3-53 | Immunoglobulin heavy variable 3-53 (Ig heavy chain V-III region BUT) | 0.31 | 0.07 |
| P02748 | C9 | Complement component C9 [Cleaved into: Complement component C9a; Complement component C9b] | 0.12 | 0.07 |
| Q06033_2 |  | Inter-alpha-trypsin inhibitor heavy chain H3 (ITI heavy chain H3) (ITI-HC3) (Inter-alpha-inhibitor heavy chain 3) (Serum-derived hyaluronan-associated protein) (SHAP) | 0.13 | 0.07 |
| F5H6I0 | B2M | Beta-2-microglobulin | 0.14 | 0.07 |
| P01871 | IGHM | Immunoglobulin heavy constant mu (Ig mu chain C region) (Ig mu chain C region BOT) (Ig mu chain C region GAL) (Ig mu chain C region OU) | 0.44 | 0.07 |
| P08571 | CD14 | Monocyte differentiation antigen CD14 (Myeloid cell-specific leucine-rich glycoprotein) (CD antigen CD14) [Cleaved into: Monocyte differentiation antigen CD14, urinary form; Monocyte differentiation antigen CD14, membrane-bound form] | 0.13 | 0.08 |
| O00391_2 |  | Sulfhydryl oxidase 1 (hQSOX) (EC 1.8.3.2) (Quiescin Q6) | 0.11 | 0.08 |
| P04206 |  | Immunoglobulin kappa variable 3-20 (Ig kappa chain V-III region B6) (Ig kappa chain V-III region GOL) (Ig kappa chain V-III region HAH) (Ig kappa chain V-III region HIC) (Ig kappa chain V-III region IARC/BL41) (Ig kappa chain V-III region NG9) (Ig kappa chain V-III region SIE) (Ig kappa chain V-III region Ti) (Ig kappa chain V-III region WOL) | 0.34 | 0.08 |
| P07225 | PROS1 | Vitamin K-dependent protein S | 0.04 | 0.08 |
| P29622 | SERPI4 | Kallistatin (Kallikrein inhibitor) (Peptidase inhibitor 4) (PI-4) (Serpin A4) | -0.16 | 0.08 |
| H0Y755 | FCGR3A | Low affinity immunoglobulin gamma Fc region receptor III-A (Fragment) | 0.18 | 0.08 |
| P10643 | C7 | Complement component C7 | 0.06 | 0.08 |
| P01859 | IGHG2 | Immunoglobulin heavy constant gamma 2 (Ig gamma-2 chain C region) (Ig gamma-2 chain C region DOT) (Ig gamma-2 chain C region TIL) (Ig gamma-2 chain C region ZIE) | 0.36 | 0.09 |
| P01625 |  | Immunoglobulin kappa variable 4-1 (Ig kappa chain V-IV region B17) (Ig kappa chain V-IV region JI) (Ig kappa chain V-IV region Len) (Ig kappa chain V-IV region STH) | 0.38 | 0.09 |
| O75636 | FCN3 | Ficolin-3 (Collagen/fibrinogen domain-containing lectin 3 p35) (Collagen/fibrinogen domain-containing protein 3) (Hakata antigen) | 0.14 | 0.09 |
| P25311 | AZGP1 | Zinc-alpha-2-glycoprotein (Zn-alpha-2-GP) (Zn-alpha-2-glycoprotein) | -0.14 | 0.09 |
| Q96PD5 | PGLYRP2 | N-acetylmuramoyl-L-alanine amidase (EC 3.5.1.28) (Peptidoglycan recognition protein 2) (Peptidoglycan recognition protein long) (PGRP-L) | -0.14 | 0.10 |
| P01019 | AGT | Angiotensinogen (Serpin A8) [Cleaved into: Angiotensin-1 (Angiotensin 1-10) (Angiotensin I) (Ang I); Angiotensin-2 (Angiotensin 1-8) (Angiotensin II) (Ang II); Angiotensin-3 (Angiotensin 2-8) (Angiotensin III) (Ang III) (Des-Asp[1]-angiotensin II); Angiotensin-4 (Angiotensin 3-8) (Angiotensin IV) (Ang IV); Angiotensin 1-9; Angiotensin 1-7; Angiotensin 1-5; Angiotensin 1-4] | 0.21 | 0.11 |
| P04040 | CAT | Catalase (EC 1.11.1.6) | 0.18 | 0.11 |
| P01860 | IGHG3 | Immunoglobulin heavy constant gamma 3 (HDC) (Heavy chain disease protein) (Ig gamma-3 chain C region) | 0.35 | 0.11 |
| P01611 | IGKV1D-12 | Immunoglobulin kappa variable 1D-12 (Ig kappa chain V-I region Wes) | 0.30 | 0.11 |
| P07195 | LDHB | L-lactate dehydrogenase B chain (LDH-B) (EC 1.1.1.27) (LDH heart subunit) (LDH-H) (Renal carcinoma antigen NY-REN-46) | 0.14 | 0.11 |
| Q9NZP8 | C1RL | Complement C1r subcomponent-like protein (C1r-LP) (C1r-like protein) (EC 3.4.21.-) (C1r-like serine protease analog protein) (CLSPa) | 0.10 | 0.11 |
| P19823 | ITIH2 | Inter-alpha-trypsin inhibitor heavy chain H2 (ITI heavy chain H2) (ITI-HC2) (Inter-alpha-inhibitor heavy chain 2) (Inter-alpha-trypsin inhibitor complex component II) (Serum-derived hyaluronan-associated protein) (SHAP) | -0.16 | 0.12 |
| P01624 | IGKV3-15 | Immunoglobulin kappa variable 3-15 (Ig kappa chain V-III region CLL) (Ig kappa chain V-III region POM) | 0.34 | 0.13 |
| P02750 | LRG1 | Leucine-rich alpha-2-glycoprotein (LRG) | 0.13 | 0.13 |
| P20851_2 |  | C4b-binding protein beta chain | 0.03 | 0.13 |
| P07360 | C8G | Complement component C8 gamma chain | 0.05 | 0.13 |
| F5GY80 | C8B | Complement component C8 beta chain | 0.04 | 0.13 |
| K7ER74 | APOC4-APOC2 | APOC4-APOC2 readthrough (NMD candidate) | 0.24 | 0.13 |
| P04406_2 |  | Glyceraldehyde-3-phosphate dehydrogenase (GAPDH) (EC 1.2.1.12) (Peptidyl-cysteine S-nitrosylase GAPDH) (EC 2.6.99.-) | 0.20 | 0.14 |
| B4E1Z4 |  | cDNA FLJ55673, highly similar to Complement factor B | 0.03 | 0.15 |
| P01011 | SERPI3 | Alpha-1-antichymotrypsin (ACT) (Cell growth-inhibiting gene 24/25 protein) (Serpin A3) [Cleaved into: Alpha-1-antichymotrypsin His-Pro-less] | 0.13 | 0.15 |
| D6RA08 |  | Deleted. | 0.12 | 0.16 |
| P00915 | CA1 | Carbonic anhydrase 1 (EC 4.2.1.1) (Carbonate dehydratase I) (Carbonic anhydrase B) (CAB) (Carbonic anhydrase I) (CA-I) | 0.10 | 0.16 |
| P02747 | C1QC | Complement C1q subcomponent subunit C | 0.12 | 0.16 |
| H0Y612 | TRIM33 | E3 ubiquitin-protein ligase TRIM33 (Fragment) | 0.15 | 0.17 |
| P07359 | GP1BA | Platelet glycoprotein Ib alpha chain (GP-Ib alpha) (GPIb-alpha) (GPIbA) (Glycoprotein Ibalpha) (Antigen CD42b-alpha) (CD antigen CD42b) [Cleaved into: Glycocalicin] | 0.06 | 0.17 |
| P02749 | APOH | Beta-2-glycoprotein 1 (APC inhibitor) (Activated protein C-binding protein) (Anticardiolipin cofactor) (Apolipoprotein H) (Apo-H) (Beta-2-glycoprotein I) (B2GPI) (Beta(2)GPI) | -0.20 | 0.18 |
| P22792 | CPN2 | Carboxypeptidase N subunit 2 (Carboxypeptidase N 83 kDa chain) (Carboxypeptidase N large subunit) (Carboxypeptidase N polypeptide 2) (Carboxypeptidase N regulatory subunit) | 0.04 | 0.19 |
| Q6EMK4 | VASN | Vasorin (Protein slit-like 2) | 0.06 | 0.19 |
| B7ZKJ8 | ITIH4 | ITIH4 protein (Inter-alpha-trypsin inhibitor heavy chain H4) | 0.11 | 0.19 |
| O14791_3 |  | Apolipoprotein L1 (Apolipoprotein L) (Apo-L) (ApoL) (Apolipoprotein L-I) (ApoL-I) | 0.08 | 0.19 |
| P02649 | APOE | Apolipoprotein E (Apo-E) | 0.16 | 0.19 |
| P55056 | APOC4 | Apolipoprotein C-IV (Apo-CIV) (ApoC-IV) (Apolipoprotein C4) | 0.15 | 0.19 |
| P05546 | SERPIND1 | Heparin cofactor 2 (Heparin cofactor II) (HC-II) (Protease inhibitor leuserpin-2) (HLS2) (Serpin D1) | -0.22 | 0.19 |
| P06276 | BCHE | Cholinesterase (EC 3.1.1.8) (Acylcholine acylhydrolase) (Butyrylcholine esterase) (Choline esterase II) (Pseudocholinesterase) | -0.19 | 0.21 |
| P48740 | MASP1 | Mannan-binding lectin serine protease 1 (EC 3.4.21.-) (Complement factor MASP-3) (Complement-activating component of Ra-reactive factor) (Mannose-binding lectin-associated serine protease 1) (MASP-1) (Mannose-binding protein-associated serine protease) (Ra-reactive factor serine protease p100) (RaRF) (Serine protease 5) [Cleaved into: Mannan-binding lectin serine protease 1 heavy chain; Mannan-binding lectin serine protease 1 light chain] | 0.04 | 0.23 |
| P20742 | PZP | Pregnancy zone protein (C3 and PZP-like alpha-2-macroglobulin domain-containing protein 6) | 0.12 | 0.25 |
| P02766 | TTR | Transthyretin (ATTR) (Prealbumin) (TBPA) | -0.18 | 0.27 |
| P32119 | PRDX2 | Peroxiredoxin-2 (EC 1.11.1.15) (Natural killer cell-enhancing factor B) (NKEF-B) (PRP) (Thiol-specific antioxidant protein) (TSA) (Thioredoxin peroxidase 1) (Thioredoxin-dependent peroxide reductase 1) | 0.02 | 0.30 |
| P01833 | PIGR | Polymeric immunoglobulin receptor (PIgR) (Poly-Ig receptor) (Hepatocellular carcinoma-associated protein TB6) [Cleaved into: Secretory component] | 0.25 | 0.30 |
| P02745 | C1QA | Complement C1q subcomponent subunit A | 0.11 | 0.31 |
| P19320_3 |  | Vascular cell adhesion protein 1 (V-CAM 1) (VCAM-1) (INCAM-100) (CD antigen CD106) | 0.13 | 0.31 |
| P22352 | GPX3 | Glutathione peroxidase 3 (GPx-3) (GSHPx-3) (EC 1.11.1.9) (Extracellular glutathione peroxidase) (Plasma glutathione peroxidase) (GPx-P) (GSHPx-P) | -0.07 | 0.32 |
| Q08380 | LGALS3BP | Galectin-3-binding protein (Basement membrane autoantigen p105) (Lectin galactoside-binding soluble 3-binding protein) (Mac-2-binding protein) (MAC2BP) (Mac-2 BP) (Tumor-associated antigen 90K) | 0.14 | 0.32 |
| P12259 | F5 | Coagulation factor V (Activated protein C cofactor) (Proaccelerin, labile factor) [Cleaved into: Coagulation factor V heavy chain; Coagulation factor V light chain] | 0.03 | 0.32 |
| P07900 | HSP90AA1 | Heat shock protein HSP 90-alpha (Heat shock 86 kDa) (HSP 86) (HSP86) (Lipopolysaccharide-associated protein 2) (LAP-2) (LPS-associated protein 2) (Renal carcinoma antigen NY-REN-38) | 0.14 | 0.34 |
| P05452 | CLEC3B | Tetranectin (TN) (C-type lectin domain family 3 member B) (Plasminogen kringle 4-binding protein) | -0.14 | 0.35 |
| P19827 | ITIH1 | Inter-alpha-trypsin inhibitor heavy chain H1 (ITI heavy chain H1) (ITI-HC1) (Inter-alpha-inhibitor heavy chain 1) (Inter-alpha-trypsin inhibitor complex component III) (Serum-derived hyaluronan-associated protein) (SHAP) | -0.13 | 0.36 |
| O43866 | CD5L | CD5 antigen-like (Apoptosis inhibitor expressed by macrophages) (hAIM) (CT-2) (IgM-associated peptide) (SP-alpha) | 0.24 | 0.37 |
| P00918 | CA2 | Carbonic anhydrase 2 (EC 4.2.1.1) (Carbonate dehydratase II) (Carbonic anhydrase C) (CAC) (Carbonic anhydrase II) (CA-II) | -0.03 | 0.37 |
| P04075 | ALDOA | Fructose-bisphosphate aldolase A (EC 4.1.2.13) (Lung cancer antigen NY-LU-1) (Muscle-type aldolase) | 0.10 | 0.39 |
| P07357 | C8A | Complement component C8 alpha chain (Complement component 8 subunit alpha) | -0.01 | 0.39 |
| H0YD13 | CD44 | CD44 antigen | -0.01 | 0.41 |
| P02751_10 | | Fibronectin (FN) (Cold-insoluble globulin) (CIG) [Cleaved into: Anastellin; Ugl-Y1; Ugl-Y2; Ugl-Y3] |  | 0.41 |
| P01877 | IGHA2 | Immunoglobulin heavy constant alpha 2 (Ig alpha-2 chain C region) (Ig alpha-2 chain C region BUT) (Ig alpha-2 chain C region LAN) | 0.32 | 0.41 |
| P01042 | KNG1 | Kininogen-1 (Alpha-2-thiol proteinase inhibitor) (Fitzgerald factor) (High molecular weight kininogen) (HMWK) (Williams-Fitzgerald-Flaujeac factor) [Cleaved into: Kininogen-1 heavy chain; T-kinin (Ile-Ser-Bradykinin); Bradykinin (Kallidin I); Lysyl-bradykinin (Kallidin II); Kininogen-1 light chain; Low molecular weight growth-promoting factor] | -0.15 | 0.42 |
| B0YIW2 | APOC3 | Apolipoprotein C-III (Apolipoprotein C-III variant 1) | 0.17 | 0.43 |
| E7EPZ9 |  | Deleted. | 0.05 | 0.43 |
| P04114 | APOB | Apolipoprotein B-100 (Apo B-100) [Cleaved into: Apolipoprotein B-48 (Apo B-48)] | 0.06 | 0.43 |
| P51884 | LUM | Lumican (Keratan sulfate proteoglycan lumican) (KSPG lumican) | -0.14 | 0.43 |
| Q5VY30 | RBP4 | Retinol-binding protein | -0.11 | 0.43 |
| Q9Y5Y7 | LYVE1 | Lymphatic vessel endothelial hyaluronic acid receptor 1 (LYVE-1) (Cell surface retention sequence-binding protein 1) (CRSBP-1) (Extracellular link domain-containing protein 1) (Hyaluronic acid receptor) | 0.14 | 0.44 |
| P04264 | KRT1 | Keratin, type II cytoskeletal 1 (67 kDa cytokeratin) (Cytokeratin-1) (CK-1) (Hair alpha protein) (Keratin-1) (K1) (Type-II keratin Kb1) | 0.18 | 0.44 |
| P04004 | VTN | Vitronectin (VN) (S-protein) (Serum-spreading factor) (V75) [Cleaved into: Vitronectin V65 subunit; Vitronectin V10 subunit; Somatomedin-B] | 0.01 | 0.46 |
| C9JF17 | APOD | Apolipoprotein D (Fragment) | -0.12 | 0.46 |
| Q16610 | ECM1 | Extracellular matrix protein 1 (Secretory component p85) | 0.03 | 0.46 |
| B1AKG0 | CFHR1 | Complement factor H-related protein 1 | 0.08 | 0.47 |
| P02679_2 |  | Fibrinogen gamma chain | 0.16 | 0.48 |
| P01591 | JCHAIN | Immunoglobulin J chain (Joining chain of multimeric IgA and IgM) | 0.21 | 0.49 |
| P35542 | SAA4 | Serum amyloid A-4 protein (Constitutively expressed serum amyloid A protein) (C-SAA) | -0.07 | 0.50 |
| P02675 | FGB | Fibrinogen beta chain [Cleaved into: Fibrinopeptide B; Fibrinogen beta chain] | 0.15 | 0.51 |
| G3V2W1 | SERPI10 | Protein Z-dependent protease inhibitor (Serpin peptidase inhibitor, clade A (Alpha-1 antiproteinase, antitrypsin), member 10, isoform CRA_a) | 0.03 | 0.51 |
| P01008 | SERPINC1 | Antithrombin-III (ATIII) (Serpin C1) | -0.11 | 0.51 |
| P60709 | ACTB | Actin, cytoplasmic 1 (Beta-actin) [Cleaved into: Actin, cytoplasmic 1, N-terminally processed] | 0.07 | 0.53 |
| P15151_3 |  | Poliovirus receptor (Nectin-like protein 5) (NECL-5) (CD antigen CD155) | 0.02 | 0.54 |
| P61626 | LYZ | Lysozyme C (EC 3.2.1.17) (1,4-beta-N-acetylmuramidase C) | 0.11 | 0.54 |
| V9GYE7 | CFHR2 | Complement factor H-related protein 2 | 0.02 | 0.54 |
| G3XAM2 | CFI | Complement factor I (Complement factor I, isoform CRA_b) | -0.03 | 0.55 |
| P69905 | HBA1 | Hemoglobin subunit alpha (Alpha-globin) (Hemoglobin alpha chain) | 0.00 | 0.55 |
| P69905 | HBA2 | Hemoglobin subunit alpha (Alpha-globin) (Hemoglobin alpha chain) | 0.00 | 0.55 |
| O95445 | APOM | Apolipoprotein M (Apo-M) (ApoM) (Protein G3a) | -0.08 | 0.55 |
| P02790 | HPX | Hemopexin (Beta-1B-glycoprotein) | -0.10 | 0.56 |
| P68871 | HBB | Hemoglobin subunit beta (Beta-globin) (Hemoglobin beta chain) [Cleaved into: LVV-hemorphin-7; Spinorphin] | 0.01 | 0.60 |
| P00747 | PLG | Plasminogen (EC 3.4.21.7) [Cleaved into: Plasmin heavy chain A; Activation peptide; Angiostatin; Plasmin heavy chain A, short form; Plasmin light chain B] | -0.09 | 0.61 |
| P00748 | F12 | Coagulation factor XII (EC 3.4.21.38) (Hageman factor) (HAF) [Cleaved into: Coagulation factor XIIa heavy chain; Beta-factor XIIa part 1; Coagulation factor XIIa light chain (Beta-factor XIIa part 2)] | 0.02 | 0.61 |
| Q92954_4 |  | Proteoglycan 4 (Lubricin) (Megakaryocyte-stimulating factor) (Superficial zone proteoglycan) [Cleaved into: Proteoglycan 4 C-terminal part] | -0.10 | 0.61 |
| P01023 | A2M | Alpha-2-macroglobulin (Alpha-2-M) (C3 and PZP-like alpha-2-macroglobulin domain-containing protein 5) | -0.06 | 0.63 |
| P06396 | GSN | Gelsolin (AGEL) (Actin-depolymerizing factor) (ADF) (Brevin) | -0.08 | 0.67 |
| P0DJI8 | SAA1 | Serum amyloid A-1 protein (SAA) [Cleaved into: Amyloid protein A (Amyloid fibril protein AA); Serum amyloid protein A(2-104); Serum amyloid protein A(3-104); Serum amyloid protein A(2-103); Serum amyloid protein A(2-102); Serum amyloid protein A(4-101)] | 0.11 | 0.68 |
| P22891 | PROZ | Vitamin K-dependent protein Z | -0.05 | 0.68 |
| P27169 | PON1 | Serum paraoxonase/arylesterase 1 (PON 1) (EC 3.1.1.2) (EC 3.1.1.81) (EC 3.1.8.1) (Aromatic esterase 1) (A-esterase 1) (K-45) (Serum aryldialkylphosphatase 1) | -0.17 | 0.69 |
| D6RF35 | GC | Vitamin D-binding protein | -0.09 | 0.69 |
| K7ERG9 | CFD | Complement factor D | 0.02 | 0.72 |
| P00742 | F10 | Coagulation factor X (EC 3.4.21.6) (Stuart factor) (Stuart-Prower factor) [Cleaved into: Factor X light chain; Factor X heavy chain; Activated factor Xa heavy chain] | 0.03 | 0.72 |
| P01024 | C3 | Complement C3 (C3 and PZP-like alpha-2-macroglobulin domain-containing protein 1) [Cleaved into: Complement C3 beta chain; C3-beta-c (C3bc); Complement C3 alpha chain; C3a anaphylatoxin; Acylation stimulating protein (ASP) (C3adesArg); Complement C3b alpha' chain; Complement C3c alpha' chain fragment 1; Complement C3dg fragment; Complement C3g fragment; Complement C3d fragment; Complement C3f fragment; Complement C3c alpha' chain fragment 2] | -0.08 | 0.72 |
| P01861 | IGHG4 | Immunoglobulin heavy constant gamma 4 (Ig gamma-4 chain C region) | 0.06 | 0.72 |
| P02042 | HBD | Hemoglobin subunit delta (Delta-globin) (Hemoglobin delta chain) | -0.03 | 0.72 |
| P02760 | AMBP | Protein AMBP [Cleaved into: Alpha-1-microglobulin (Protein HC) (Alpha-1 microglycoprotein) (Complex-forming glycoprotein heterogeneous in charge); Inter-alpha-trypsin inhibitor light chain (ITI-LC) (Bikunin) (EDC1) (HI-30) (Uronic-acid-rich protein); Trypstatin] | -0.10 | 0.72 |
| P02765 | AHSG | Alpha-2-HS-glycoprotein (Alpha-2-Z-globulin) (Ba-alpha-2-glycoprotein) (Fetuin-A) [Cleaved into: Alpha-2-HS-glycoprotein chain A; Alpha-2-HS-glycoprotein chain B] | -0.07 | 0.72 |
| P08185 | SERPI6 | Corticosteroid-binding globulin (CBG) (Serpin A6) (Transcortin) | 0.00 | 0.72 |
| P0C0L5 | C4B | Complement C4-B (Basic complement C4) (C3 and PZP-like alpha-2-macroglobulin domain-containing protein 3) [Cleaved into: Complement C4 beta chain; Complement C4-B alpha chain; C4a anaphylatoxin; C4b-B; C4d-B; Complement C4 gamma chain] | -0.01 | 0.72 |
| P0C0L5 | C4B_2 | Complement C4-B (Basic complement C4) (C3 and PZP-like alpha-2-macroglobulin domain-containing protein 3) [Cleaved into: Complement C4 beta chain; Complement C4-B alpha chain; C4a anaphylatoxin; C4b-B; C4d-B; Complement C4 gamma chain] | -0.01 | 0.72 |
| Q9UGM5 | FETUB | Fetuin-B (16G2) (Fetuin-like protein IRL685) (Gugu) | -0.12 | 0.72 |
| B4E2S7 |  | cDNA FLJ58780, highly similar to Homo sapiens lysosomal-associated membrane protein 2 (LAMP2), transcript variant LAMP2B, mRNA | 0.00 | 0.74 |
| E9PFZ2 | CP | Ceruloplasmin | 0.11 | 0.74 |
| F5GXS5 |  | Deleted. | 0.04 | 0.74 |
| P14151 | SELL | L-selectin (CD62 antigen-like family member L) (Leukocyte adhesion molecule 1) (LAM-1) (Leukocyte surface antigen Leu-8) (Leukocyte-endothelial cell adhesion molecule 1) (LECAM1) (Lymph node homing receptor) (TQ1) (gp90-MEL) (CD antigen CD62L) | 0.01 | 0.74 |
| P36955 | SERPINF1 | Pigment epithelium-derived factor (PEDF) (Cell proliferation-inducing gene 35 protein) (EPC-1) (Serpin F1) | 0.00 | 0.74 |
| Q04756 | HGFAC | Hepatocyte growth factor activator (HGF activator) (HGFA) (EC 3.4.21.-) [Cleaved into: Hepatocyte growth factor activator short chain; Hepatocyte growth factor activator long chain] | -0.02 | 0.74 |
| P05543 | SERPI7 | Thyroxine-binding globulin (Serpin A7) (T4-binding globulin) | -0.08 | 0.77 |
| F8W1Q3 |  | Biotinidase (Biotinase) (EC 3.5.1.12) | -0.01 | 0.78 |
| O75882_3 |  | Attractin (DPPT-L) (Mahogany homolog) | -0.02 | 0.78 |
| P05155 | SERPING1 | Plasma protease C1 inhibitor (C1 Inh) (C1Inh) (C1 esterase inhibitor) (C1-inhibiting factor) (Serpin G1) | 0.02 | 0.78 |
| Q96IY4 | CPB2 | Carboxypeptidase B2 (EC 3.4.17.20) (Carboxypeptidase U) (CPU) (Plasma carboxypeptidase B) (pCPB) (Thrombin-activable fibrinolysis inhibitor) (TAFI) | -0.04 | 0.78 |
| P07996 | THBS1 | Thrombospondin-1 (Glycoprotein G) | -0.08 | 0.79 |
| P08603 | CFH | Complement factor H (H factor 1) | 0.01 | 0.79 |
| P10909_4 |  | Clusterin (Aging-associated gene 4 protein) (Apolipoprotein J) (Apo-J) (Complement cytolysis inhibitor) (CLI) (Complement-associated protein SP-40,40) (Ku70-binding protein 1) (NA1/NA2) (Sulfated glycoprotein 2) (SGP-2) (Testosterone-repressed prostate message 2) (TRPM-2) [Cleaved into: Clusterin beta chain (ApoJalpha) (Complement cytolysis inhibitor a chain); Clusterin alpha chain (ApoJbeta) (Complement cytolysis inhibitor b chain)] | -0.04 | 0.82 |
| I3L145 | SHBG | Sex hormone-binding globulin (Sex hormone-binding globulin, isoform CRA_a) | -0.08 | 0.83 |
| K7ERI9 | APOC1 | Apolipoprotein C-I (Fragment) | 0.09 | 0.83 |
| P00736 | C1R | Complement C1r subcomponent (EC 3.4.21.41) (Complement component 1 subcomponent r) [Cleaved into: Complement C1r subcomponent heavy chain; Complement C1r subcomponent light chain] | -0.05 | 0.83 |
| P02775 | PPBP | Platelet basic protein (PBP) (C-X-C motif chemokine 7) (Leukocyte-derived growth factor) (LDGF) (Macrophage-derived growth factor) (MDGF) (Small-inducible cytokine B7) [Cleaved into: Connective tissue-activating peptide III (CTAP-III) (LA-PF4) (Low-affinity platelet factor IV); TC-2; Connective tissue-activating peptide III(1-81) (CTAP-III(1-81)); Beta-thromboglobulin (Beta-TG); Neutrophil-activating peptide 2(74) (NAP-2(74)); Neutrophil-activating peptide 2(73) (NAP-2(73)); Neutrophil-activating peptide 2 (NAP-2); TC-1; Neutrophil-activating peptide 2(1-66) (NAP-2(1-66)); Neutrophil-activating peptide 2(1-63) (NAP-2(1-63))] | -0.08 | 0.83 |
| Q13103 | SPP2 | Secreted phosphoprotein 24 (Spp-24) (Secreted phosphoprotein 2) | -0.02 | 0.83 |
| P0C0L4 | C4A | Complement C4-A (Acidic complement C4) (C3 and PZP-like alpha-2-macroglobulin domain-containing protein 2) [Cleaved into: Complement C4 beta chain; Complement C4-A alpha chain; C4a anaphylatoxin; C4b-A; C4d-A; Complement C4 gamma chain] | 0.02 | 0.84 |
| E9PGP2 |  | Deleted. | 0.03 | 0.85 |
| P15169 | CPN1 | Carboxypeptidase N catalytic chain (CPN) (EC 3.4.17.3) (Anaphylatoxin inactivator) (Arginine carboxypeptidase) (Carboxypeptidase N polypeptide 1) (Carboxypeptidase N small subunit) (Kininase-1) (Lysine carboxypeptidase) (Plasma carboxypeptidase B) (Serum carboxypeptidase N) (SCPN) | -0.01 | 0.85 |
| P08697 | SERPINF2 | Alpha-2-antiplasmin (Alpha-2-AP) (Alpha-2-plasmin inhibitor) (Alpha-2-PI) (Serpin F2) | 0.01 | 0.85 |
| H0YAC1 | KLKB1 | Plasma kallikrein (Fragment) | -0.04 | 0.90 |
| P00740_2 |  | Coagulation factor IX (EC 3.4.21.22) (Christmas factor) (Plasma thromboplastin component) (PTC) [Cleaved into: Coagulation factor IXa light chain; Coagulation factor IXa heavy chain] | -0.01 | 0.90 |
| P04180 | LCAT | Phosphatidylcholine-sterol acyltransferase (EC 2.3.1.43) (Lecithin-cholesterol acyltransferase) (Phospholipid-cholesterol acyltransferase) | -0.01 | 0.90 |
| P00739 | HPR | Haptoglobin-related protein | 0.03 | 0.93 |
| P02743 | APCS | Serum amyloid P-component (SAP) (9.5S alpha-1-glycoprotein) [Cleaved into: Serum amyloid P-component(1-203)] | 0.03 | 0.94 |
| P09871 | C1S | Complement C1s subcomponent (EC 3.4.21.42) (C1 esterase) (Complement component 1 subcomponent s) [Cleaved into: Complement C1s subcomponent heavy chain; Complement C1s subcomponent light chain] | -0.08 | 1.00 |
| P00450 | CP | Ceruloplasmin (EC 1.16.3.1) (Ferroxidase) | -0.05 | 1.00 |
| P00734 | F2 | Prothrombin (EC 3.4.21.5) (Coagulation factor II) [Cleaved into: Activation peptide fragment 1; Activation peptide fragment 2; Thrombin light chain; Thrombin heavy chain] | -0.05 | 1.00 |
| P02774 | GC | Vitamin D-binding protein (DBP) (VDB) (Gc protein-derived macrophage activating factor) (Gc-MAF) (GcMAF) (Gc-globulin) (Group-specific component) (Gc) (Vitamin D-binding protein-macrophage activating factor) (DBP-maf) | -0.06 | 1.00 |
| P04217 | A1BG | Alpha-1B-glycoprotein (Alpha-1-B glycoprotein) | -0.02 | 1.00 |
| P06727 | APOA4 | Apolipoprotein A-IV (Apo-AIV) (ApoA-IV) (Apolipoprotein A4) | 0.00 | 1.00 |
| P13671 | C6 | Complement component C6 | -0.05 | 1.00 |
| P43652 | AFM | Afamin (Alpha-albumin) (Alpha-Alb) | -0.07 | 1.00 |
| P69891 | HBG1 | Hemoglobin subunit gamma-1 (Gamma-1-globin) (Hb F Agamma) (Hemoglobin gamma-1 chain) (Hemoglobin gamma-A chain) | -0.07 | 1.00 |

*log fold change - positive value for log fold change indicate higher enrichment in HIV(+); °false-discovery rate adjusted after logistic regression using inverse probability weighting accounting for recruitment site, sex , age, HIV status, mid-upper arm circumference, and presence of oedema

Supplementary Figure 1.


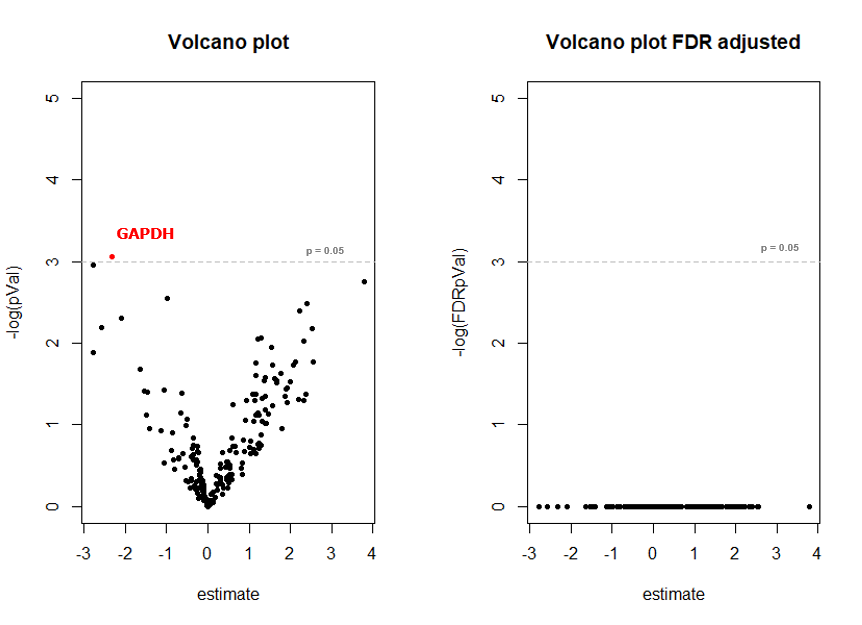


Volcano plot showing protein differential analysis among HIV(+) children on highly active antiretroviral therapy (HAART) (n = 14) and ART naïve (n = 27). Shown are results for both raw p values (left plot) and false discovery rate (Benjamini-Hochberg method) adjusted p values (right plot)
